# Behavior of Callers to a Crisis Helpline Before and During the COVID-19 Pandemic: Quantitative Data Analysis

**DOI:** 10.2196/22984

**Published:** 2020-11-06

**Authors:** Robin Turkington, Maurice Mulvenna, Raymond Bond, Edel Ennis, Courtney Potts, Ciaran Moore, Louise Hamra, Jacqui Morrissey, Mette Isaksen, Elizabeth Scowcroft, Siobhan O'Neill

**Affiliations:** 1 School of Computing Ulster University Newtownabbey United Kingdom; 2 School of Psychology Ulster University Coleraine United Kingdom; 3 Samaritans Ireland Dublin Ireland; 4 Samaritans UK Ewell United Kingdom

**Keywords:** COVID-19, coronavirus, pandemic, mental health, crisis helplines, machine learning, clustering, caller behavior

## Abstract

**Background:**

The World Health Organization declared the outbreak of COVID-19 to be an international pandemic in March 2020. While numbers of new confirmed cases of the disease and death tolls are rising at an alarming rate on a daily basis, there is concern that the pandemic and the measures taken to counteract it could cause an increase in distress among the public. Hence, there could be an increase in need for emotional support within the population, which is complicated further by the reduction of existing face-to-face mental health services as a result of measures taken to limit the spread of the virus.

**Objective:**

The objective of this study was to determine whether the COVID-19 pandemic has had any influence on the calls made to Samaritans Ireland, a national crisis helpline within the Republic of Ireland.

**Methods:**

This study presents an analysis of calls made to Samaritans Ireland in a four-week period before the first confirmed case of COVID-19 (calls=41,648, callers=3752) and calls made to the service within a four-week period after a restrictive lockdown was imposed by the government of the Republic of Ireland (calls=46,043, callers=3147). Statistical analysis was conducted to explore any differences between the duration of calls in the two periods at a global level and at an hourly level. We performed k-means clustering to determine the types of callers who used the helpline based on their helpline call usage behavior and to assess the impact of the pandemic on the caller type usage patterns.

**Results:**

The analysis revealed that calls were of a longer duration in the postlockdown period in comparison with the pre–COVID-19 period. There were changes in the behavior of individuals in the cluster types defined by caller behavior, where some caller types tended to make longer calls to the service in the postlockdown period. There were also changes in caller behavior patterns with regard to the time of day of the call; variations were observed in the duration of calls at particular times of day, where average call durations increased in the early hours of the morning.

**Conclusions:**

The results of this study highlight the impact of COVID-19 on a national crisis helpline service. Statistical differences were observed in caller behavior between the prelockdown and active lockdown periods. The findings suggest that service users relied on crisis helpline services more during the lockdown period due to an increased sense of isolation, worsening of underlying mental illness due to the pandemic, and reduction or overall removal of access to other support resources. Practical implications and limitations are discussed.

## Introduction

COVID-19 has spread globally; by May 1, 2020, the disease had reached over 215 countries and territories worldwide, with over 3.1 million confirmed cases and 224,172 confirmed deaths [1]. On January 30, 2020, the World Health Organization (WHO) declared the COVID-19 outbreak to be a Public Health Emergency of International Concern (PHEIC); this was only the sixth time the WHO had declared a PHEIC since 2005. Since this declaration, many governments have imposed lockdown measures preventing people from mixing and attending work or school and have instructed the general public to adhere to “social distancing” or self-isolate to slow the spread of the disease. There is concern that COVID-19 has had a negative impact on the mental well-being of individuals, particularly with the implementation of strict lockdown measures.

Data indicate that symptoms of anxiety and depression increased as a result of the pandemic and peaked at the time of government announcements regarding restrictions to curb the spread of the virus [2,3]. Furthermore, evidence indicates that the people at highest risk of having symptoms of mental illness are those in lower income households, who are required to self-isolate because of their risk of having an adverse outcome if they contract the virus, and who have existing mental health problems [2]. Although the public relies on news and media reports to assess the evolving state of the crisis, repeated exposure to negative news coverage of the pandemic can enhance psychological distress, leading to the development and worsening of mental illness symptoms [4,5]. The need to self-isolate may cause people to feel isolated from friends and family, and the impact is greater for people with underlying psychological vulnerability or mental illness who rely on social and support networks to stay well. Moreover, many mental health services and the availability of face-to-face support were disrupted as a result of the restrictions, leaving people who depend on such services without the support groups and resources that benefit them [6]. Some individuals may be reluctant to seek help and support from face-to-face mental health services due to concern that such services are being overwhelmed or out of fear of contracting COVID-19 in a face-to-face appointment setting [5]. These individuals may therefore rely more on help from additional remote services, such as suicide prevention and crisis helplines [6,7].

Suicide prevention and crisis helplines provide support to people who are experiencing a crisis, which is defined as a state of psychological disequilibrium where the individual’s coping mechanisms are no longer effective [8,9]. Samaritans Ireland is a crisis support and suicide prevention helpline that provides free confidential support to people, many of whom are highly distressed, suicidal, and may have underlying mental illnesses [10]. Callers to Samaritans speak to trained volunteers who provide respectful and nonjudgmental active listening. Callers may contact the service on a single occasion or repeatedly; for a systematic review on repeat callers, see Middleton et al [11]. Samaritans is one of the oldest helplines in operation within the United Kingdom and Ireland, and it provides free confidential support 24 hours per day every day of the year [10].

Understanding a caller’s needs based on contact patterns and how they interact with the service can be valuable for operational purposes and for understanding how suicide prevention and crisis helplines can be used in population-level well-being and suicide prevention programs [12-14]. Patterns of calls to crisis or suicide prevention helplines may also reflect the impact of COVID-19 on suicidal distress, mental health, and well-being. An understanding of caller patterns will also help inform population-level support planning and the guidance provided regarding accessing emotional support. Crisis lines have been identified as an important means of supporting people who are at risk of mental illness in a time where face-to-face contact must be avoided [15]. It is therefore important to examine patterns of help seeking and use of crisis helplines so that services can respond accordingly.

**Figure 1:**
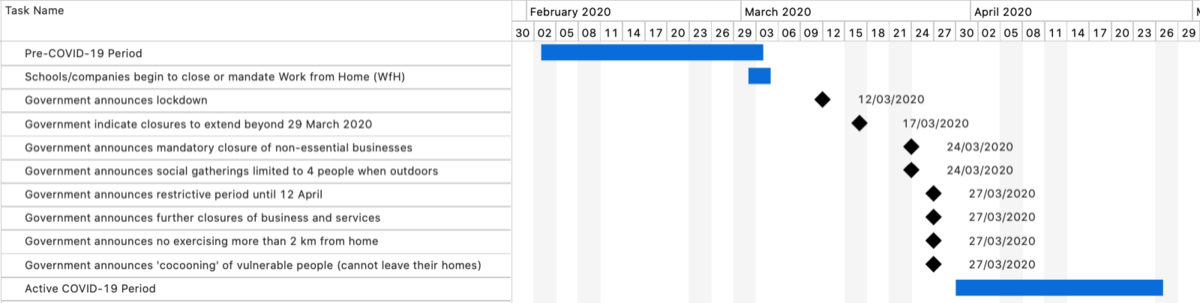
Timelines of the Pre–COVID-19 Period, dates of key government messages, and the Active COVID-19 Period.

The objective of the current study is to analyze the potential impact of the COVID-19 pandemic on the behavior of callers to Samaritans Ireland. The study analyzed caller behavior from a four-week period prior and up to the first confirmed case of COVID-19 in the Republic of Ireland and compared it to caller behavior from a similar four-week period after the introduction of the lockdown restrictions by the Irish government (Figure 1).

This research addresses the following research questions:

- Which aspects of caller behavior have changed as a result of the COVID-19 pandemic?
- Which cohorts of callers demonstrate changes in behavior in response to the restrictions imposed to address the pandemic?

## Methods

### Data Background

Calls made to Samaritans Ireland are made from, and answered, within the Republic of Ireland. Each call that is made to the service is represented electronically. The fields that were used for the analysis were the caller identifier (which was modified to anonymize the caller); the date and time stamp of the call; and the duration of the call (in seconds).

Call detail records were retrieved from a dedicated application programming interface (API) created by Samaritans. This enabled the creation of a real-time anonymized data stream for analysis. There are no identifiable aspects within the call data, nor are there any data fields that contain complementary information about the caller’s condition or any indications of whether the caller is living with any physical or mental illness, if they are already a service user, the level of distress the caller is in, or whether the caller is experiencing a crisis at the time of the call.

### Analysis of Key Dates in the Timeline Before and After the COVID-19 Lockdown

Using the dedicated API, call data from January 1, 2019, to May 11, 2020, were retrieved, which equated to 1,054,089 calls by 30,659 callers. After the data were subjected to data cleansing (ie, normalization of time and date stamps, inspection and removal of anomalous data entries), two new call data frame subsets were derived from the original data set. One of these new data frames consisted of all calls that were made to the service within a four-week period prior to the first confirmed case of COVID-19 in the Republic of Ireland (Week 6 to Week 9 of 2020; calls=41,648, callers=3752); this is referred to as the Pre–COVID-19 period. The other new data frame consisted of all calls that were made to the service within a four-week period after the commencement of the lockdown (Week 14 to Week 17 of 2020; (calls=46,043, callers=3147); this is referred to as the Active COVID-19 period.

### Unsupervised Machine Learning Using k-Means Clustering

The call data were subjected to k-means clustering to discover the types of callers that used the service. In k-means clustering, data points are grouped together based on their closeness by Euclidean distance. In other words, the aim is to find *k* groups in *n* objects based on the similarity of their characteristics, where the characteristics in one group show high similarity with each other but low similarity with other groups [16,17]. To determine the types of callers that use the service, three attributes of caller behavior were selected for clustering: the number of calls made by each caller, the mean duration of the calls by each caller, and the standard deviation of the duration of the calls made by each caller.

These attributes of caller behavior were chosen due to their explanatory power; the number of calls that the caller makes to the service indicates the frequency of help-seeking, the average duration of the calls indicates how complex the calls may be, and the standard deviation of the call duration indicates the consistency (or inconsistency) of the call durations. New data sets that contained numerical summaries of these attributes for callers in each period were created. Each attribute was then scaled for standardization, which is an appropriate prerequisite for k-means clustering. The next stage was to specify the value of *k,* which specifies the number of groups into which the data are to be clustered. Based on previous research that used k-means clustering to identify caller types [17-19], *k* was set to 5, meaning that 5 caller types were discovered as a result of clustering.

The 5 caller types can be described as follows:

1. Typical callers: These callers make approximately 5 calls on average to the helpline. Calls last approximately 5 minutes on average and are consistent in duration; this group is the largest in size.
2. High Frequency callers: These callers make the most calls on average to the helpline, averaging hundreds of calls. Calls are very short in duration but can be highly variable; this group is the smallest in size.
3. Regular callers: These callers make the second highest average number of calls to the service. They can make upwards of a hundred calls to the service on average; however, this number can be greater or smaller depending on the period of the data set being analyzed. Calls can last approximately 10 minutes on average, although the duration of the calls may be much longer or shorter.
4. Unpredictable callers: These callers make approximately 8-12 calls on average. Calls can be upwards of 25 minutes long; however, the call duration is the most variable of all the cluster types.
5. Single Lengthy callers: These callers make 1 to 2 calls on average. The call duration is the longest and most consistent of all the caller types.

Clustering was conducted on both the Pre–COVID-19 and Active COVID-19 periods, and any changes in caller *archetypes* (cluster types defined by caller behavior) will be discussed.

### Data Analytics Materials

R 3.5.1 (the R Project) was used in all aspects of analysis. The ggplot2 package [20] was used to create data visualizations, while base R functions were used to conduct k-means clustering analysis on the call data and other statistical analyses. The unpaired Wilcoxon rank sum test was conducted to compare differences in each hourly mean duration between the Pre–COVID-19 and Active COVID-19 periods.

## Results

### Differences in Call Duration Between the Pre–COVID-19 and Active COVID-19 Periods

**Figure 2:**
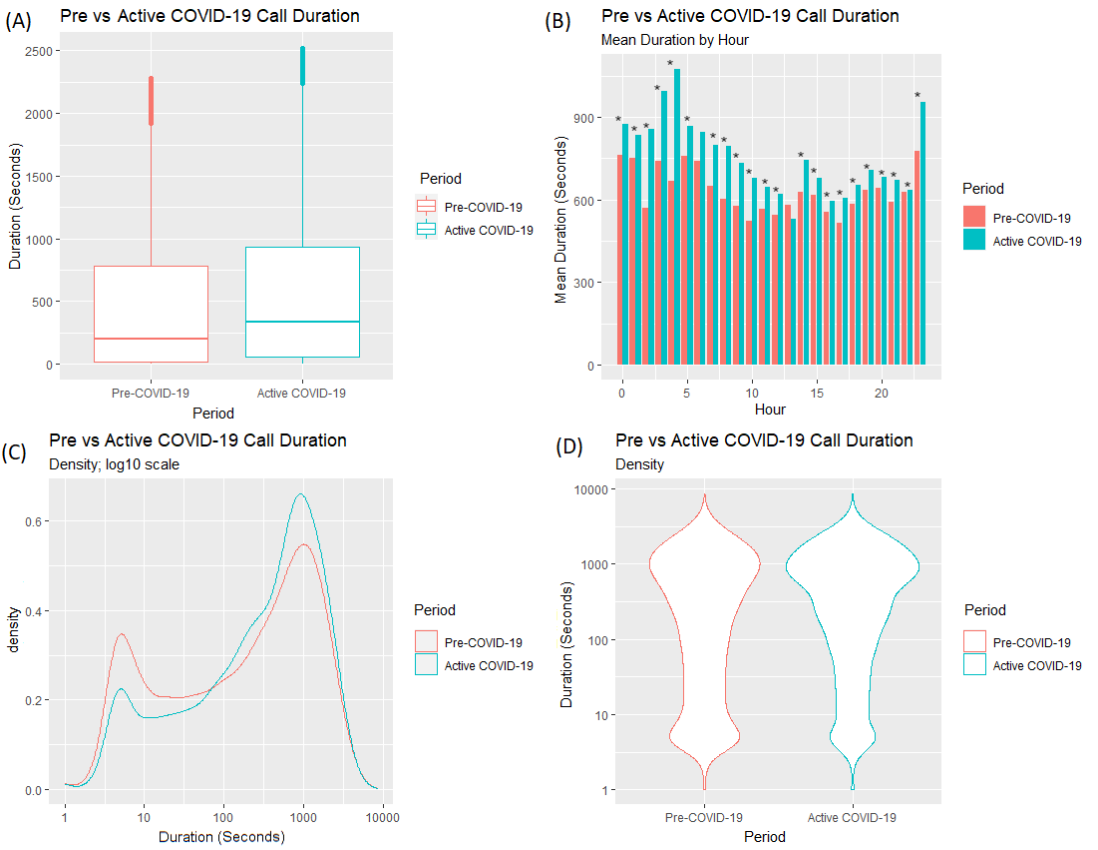
Differences in (A) call duration for answered calls between the Pre–COVID-19 and Active COVID-19 periods; (B) mean duration of calls by hour of day; and (C,D) density of call duration.

There was an increase in the mean and median duration of calls by hour of day across all but one hour from the Pre–COVID-19 period (Figure 2A and Figure 2B; mean=620 seconds/10.33 minutes; median=250 seconds/4.17 minutes) to the Active COVID-19 period (mean=709 seconds/11.82 minutes; median=388 seconds/6.47 minutes); a *t* test found a significant difference (t_51434_=11.94, *P*<.001) in call duration between the Pre–COVID-19 period and the Active COVID-19 period. An unpaired Wilcoxon rank sum test was conducted to compare differences between each hourly mean duration between the Pre–COVID-19 and Active COVID-19 periods; 22 of the 24 hours yielded a statistically significant difference between the average durations in both periods. There was a lower density (the term *density* refers to the distribution of calls over a continuous interval; in other words, the distribution of calls based on their duration) of calls with a shorter duration from the Pre–COVID-19 period to the Active COVID-19 period (Figure 2C and Figure 2D). In contrast, there was an increase in the density of calls of a longer duration from the Pre–COVID-19 period to the Active COVID-19 period.

**Figure 3:**
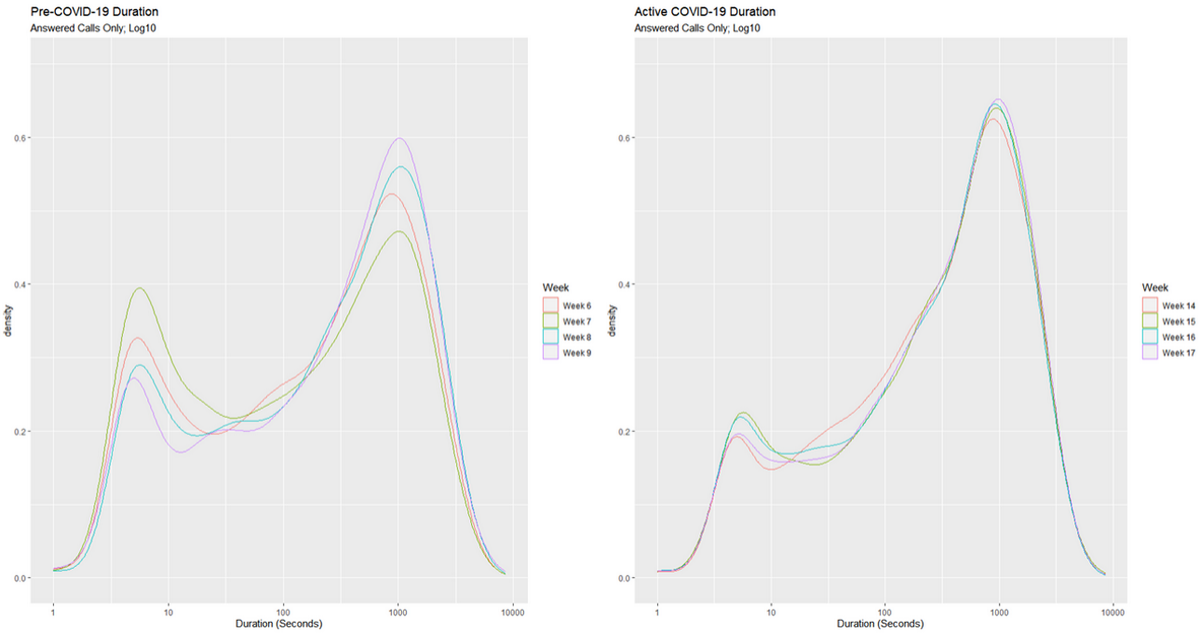
Densities of call durations of answered calls by week within the Pre–COVID-19 period (left) and Active COVID-19 period (right).

Figure 3 displays the changes in the density of call durations for answered calls across each of the consecutive weeks in the Pre–COVID-19 and Active COVID-19 periods. Within the Pre–COVID-19 period, there was a considerable amount of variation in call duration density across the weeks. In the Active COVID-19 period, the distribution of the data appeared to be stable across Week 14, Week 15, Week 16, and Week 17.

Compared to the Pre–COVID-19 period, all four Active COVID-19 weeks display a lower density of calls with short durations, with Week 14 displaying the lowest density of calls with short durations and Week 15 displaying the highest. There was little variation in the density of calls with long durations between Week 14, Week 15, and Week 16; all four Active COVID-19 weeks show a higher density of calls with longer durations than all the Pre–COVID-19 weeks. The same analysis was conducted on calls made in comparative weeks in 2019. A similar bimodal profile was found; however, the trends in Weeks 14-17 in 2019 were not as significant as those observed in the Active COVID-19 weeks in 2020. In 2019, the percentage increase in long calls (ie, calls over 600 seconds/10 minutes in duration) from the Pre–COVID-19 period to the Active COVID-19 period was 2.1; in 2020, the percentage increase in long calls from the Pre–COVID-19 period to the Active COVID-19 period was 6.1.

A one-way test was conducted to determine if there were any significant differences in the duration of calls between the weeks within both periods. In the Pre–COVID-19 period, there were significant differences across the four weeks (F_3,15528_=50.19, *P*<.001; Table 1). In the Active COVID-19 period, there were no significant differences across the four weeks (F_3,13544_=2.1026, *P*=.09; Table 2).

**Table 1:** Statistical comparisons between weeks in the Pre–COVID-19 period.

Period	Difference	Confidence interval	*t* value	Degrees of freedom	*P* value	Adjusted *P* value
Week 7-Week 6	–51	–85to –17	3.88	15,081	<.001	<.001
Week 8-Week 6	84	48 to 121	5.93	14,165	<.001	<.001
Week 9-Week 6	97	59 to 135	6.6	12,968	<.001	<.001
Week 8-Week 7	136	100 to 171	9.75	14,183	<.001	<.001
Week 9-Week 7	148	111 to 186	10.3	12,808	<.001	<.001
Week 9-Week 8	13	–27 to 52	0.83	13,099	.84	.84

**Table 2:** Statistical comparisons between weeks in the Active COVID-19 period.

Period	Difference	Confidence interval	*t* value	Degrees of freedom	*P* value	Adjusted *P* value
Week 15-Week 14	18.7	–21.7 to 59	1.19	12,159	.63	.95
Week 16-Week 14	–3.3	–42.7 to 36	0.21	12,247	>.99	>.99
Week 17-Week 14	30.5	–10 to 71	1.93	11,951	.21	.64
Week 16-Week 15	–22	–61.6 to 18	1.43	12,405	.48	.95
Week 17-Week 15	11.7	–28.9 to 52	0.74	12,101	.88	>.99
Week 17-Week 16	33.8	–5.8 to 73	2.19	12,166	.13	.64

### Clustering Analysis: Differences in Caller Characteristics Between the Pre–COVID-19 and Active COVID-19 Period

Table 3 and Table 4 show the cluster characteristics of the callers who contacted the service within the Pre–COVID-19 period and Active COVID-19 period, respectively. Some notable fluctuations in cluster means are noticeable in the High Frequency caller clusters in relation to the number of calls made to the service. Otherwise, the cluster centroids remain stable between the Pre–COVID-19 and Active COVID-19 periods.

**Table 3:** Cluster centroids of callers within the Pre–COVID-19 period.

Caller type	Average number of calls	Mean duration	Standard deviation of duration	Cluster size	Within sum of squares
Typical	3.685639	213.3336	48.00642	2284	501.3016
High Frequency	424.8	294.3486	419.1129	35	868.5136
Regular	22.039326	675.4679	725.19471	712	658.9827
Unpredictable	9.686636	1586.4138	1626.68238	217	430.4528
Single Lengthy	1.126984	1977.2117	33.53513	504	600.5583

**Table 4:** Cluster centroids of callers within the Active COVID-19 period.

Caller type	Average number of calls	Mean duration	Standard deviation of duration	Cluster size	Within sum of squares
Typical	3.953930	183.4235	48.05530	1845	379.3097
High Frequency	396.113636	314.5596	456.93319	44	694.0469
Regular	26.471942	655.0175	658.17619	695	576.7514
Unpredictable	12.257282	1336.8626	1493.99101	206	328.7420
Single Lengthy	1.109244	1950.0461	25.32212	357	568.8687

**Figure 4:**
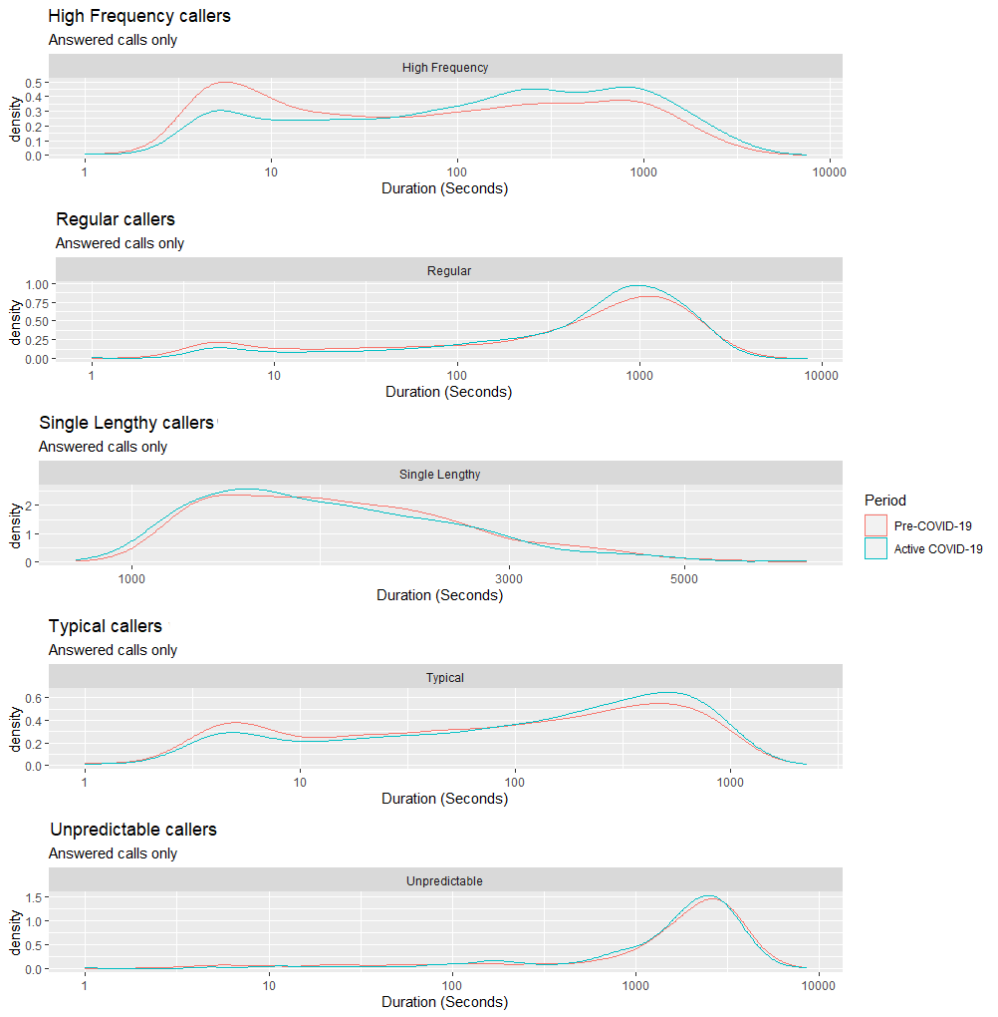
Densities of call durations for the five caller types from the Pre–COVID-19 period to the Active COVID-19 period.

Figure 4 displays the distributions of the call duration data for the five caller archetypes. The five caller types show similar distributions from the Pre–COVID-19 period to the Active COVID-19 period, with some changes for Typical callers, High Frequency callers, and Single Lengthy callers. Typical callers begin to trend toward longer calls while showing a reduced density of calls with shorter durations; a similar trend is also noticeable with High Frequency Callers.

Single Lengthy callers exhibited a higher density of call frequency, with longer call durations in the Active COVID-19 period compared to the Pre–COVID-19 period. The Wilcox ranked sum test was conducted to determine whether the caller types differed in call duration between periods. Statistically significant differences were observed within the Typical caller type (W=709173, *P*<.001), High Frequency caller type (W=42237518, *P*<.001), and Regular caller type (W=49030484, *P*<.001).

**Figure 5:**
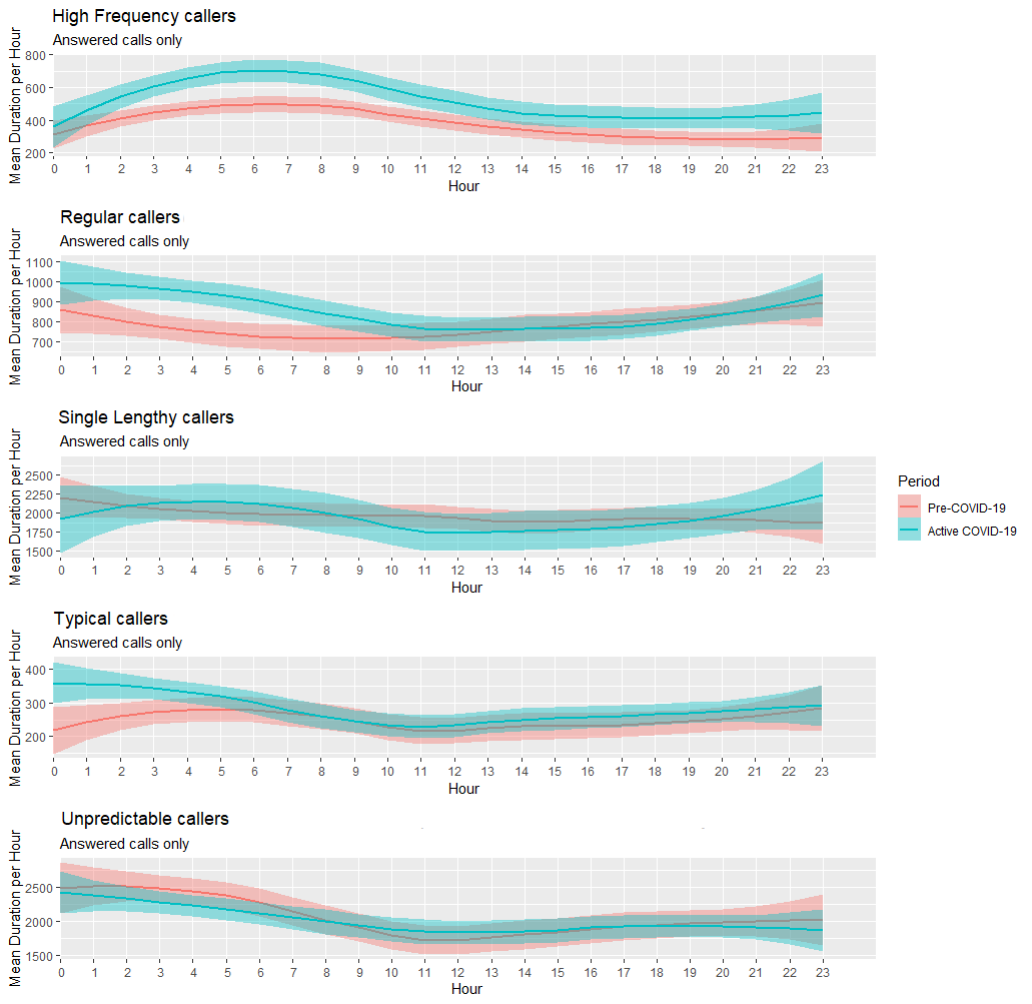
Smoothed conditional means plots displaying the mean call durations in seconds for the five caller types across a 24-hour period between the Pre-COVID-19 and Active COVID-19 periods.

Figure 5 displays the differences in the mean durations at each hour of the day at the cluster level. The Wilcox ranked sum test was conducted to compare the differences in mean duration (in seconds) at each hourly interval from the Pre–COVID-19 period to the Active COVID-19 period for each cluster.

There were visible differences in mean call durations by hour between the Pre–COVID-19 and Active COVID-19 periods for High Frequency callers. In contrast, Single Lengthy and Unpredictable callers showed similar patterns during both periods.

### Common Callers

This phase of analysis focuses on the callers who contacted the service in both the Pre–COVID-19 period and the Active COVID-19 period, termed *common callers*.

**Figure 6:**
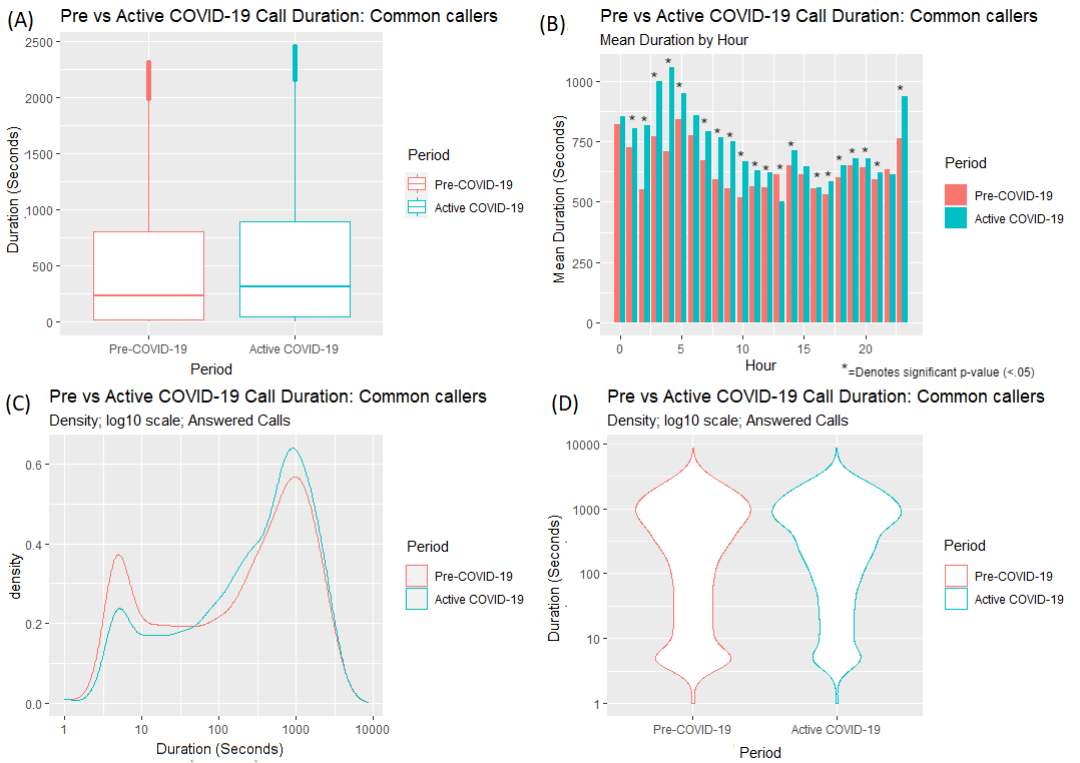
Differences in (A) call duration for answered calls by common callers in the Pre–COVID-19 and Active COVID-19 periods; (B) mean duration of calls by hour of day; and (C,D) density of call duration.

Figure 6 displays the distributions of the call duration of answered calls for callers who contacted the service in both the Pre–COVID-19 and Active COVID-19 periods. The mean and median call duration (Figure 6A) increased from the Pre–COVID-19 period (mean=628 seconds/10.47 minutes; median=279 seconds/4.65 minutes) to the Active COVID-19 period (mean=689 seconds/11.48 minutes; median=360 seconds/6 minutes); a *t* test found a significant difference between periods with regards to call duration (t_40657_=–7.2291, *P*<.001). Similar to the findings regarding call duration from all callers (Figure 2C and 2D), the density of call durations for shorter calls decreased from the Pre–COVID-19 period to the Active COVID-19 period, while the density of call durations for longer calls increased from the Pre–COVID-19 period to the Active COVID-19 period (Figure 6C and Figure 6D). The same analysis was conducted on common callers within the comparative weeks in 2019. In 2019, the percentage increase in long calls (ie, calls over 600 seconds/10 minutes) from the Pre–COVID-19 period to the Active COVID-19 period was 2.1%; in 2020, the percentage increase in long calls from the Pre–COVID-19 period to Active COVID-19 period was 4%.

**Figure 7:**
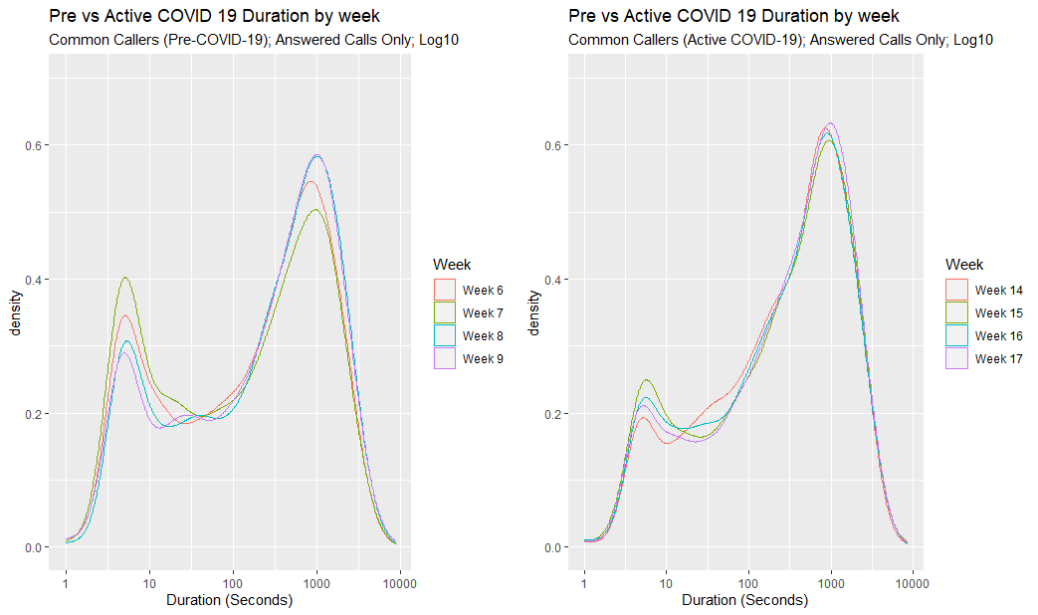
Density of call durations for answered calls by each week by common callers in the Pre–COVID-19 period (left) and Active COVID-19 period (right).

Figure 7 displays the changes in the density of call durations for answered calls across each of the consecutive weeks for the callers who contacted the service in both the Pre–COVID-19 and Active COVID-19 periods. There was a similar trend in the density of call durations for all callers (Figure 3). Within the Pre–COVID-19 period, we observed a variation in call duration density across the weeks.

In the Active COVID-19 period, there was little variation in call duration density across the weeks. Compared to the Pre–COVID-19 period, all four Active COVID-19 weeks displayed a lower density of calls with shorter call durations in total. The density of call durations was also analyzed for the same weeks in 2019, and while a similar trend in the density of call durations was observed, the trend was not as significant as those observed in the 2020 weeks.

A one-way test showed that in the Pre–COVID-19 period, there were significant differences across the four weeks (F_3,12285_=28.8, *P*<.001; Table 5). In the Active COVID-19 period, there were no significant differences across the four weeks (F_3,10726_=1.8009, *P*=.14; Table 6).

**Table 5:** Comparisons between weeks in the Pre–COVID-19 period (2020) for common callers.

Period	Difference	Confidence interval	*t* value	Degrees of freedom	*P* value	Adjusted *P* value
Week 7-Week 6	–25.2	–64 to 13	1.68	11,466	.34	.4
Week 8-Week 6	91.6	51 to 133	5.75	10,923	<.001	<.001
Week 9-Week 6	88.9	47 to 131	5.47	10,635	<.001	<.001
Week 8-Week 7	116.8	76 to 157	7.43	11,121	<.001	<.001
Week 9-Week 7	114.1	73 to 155	7.11	10,766	<.001	<.001
Week 9-Week 8	–2.7	–46 to 41	0.16	10,719	>.99	>.99

**Table 6:** Comparisons between weeks in the Active COVID-19 period (2020) for common callers.

Period	Difference	Confidence interval	*t* value	Degrees of freedom	*P* value	Adjusted *P* value
Week 15-Week 14	13.5	–31.1 to 58	0.78	9607	.86	>.99
Week 16-Week 14	2.5	–41 to 46	0.15	9815	>.99	>.99
Week 17-Week 14	36.4	–8.4 to 81	2.09	9407	.16	.61
Week 16-Week 15	–11	–55.4 to 33	0.64	9854	.92	>.99
Week 17-Week 15	–22.9	–22.7 to 69	1.29	9504	.57	>.99
Week 17-Week 16	34	–10.6 to 78	1.96	9634	.20	.61

### New Callers

**Figure 8:**
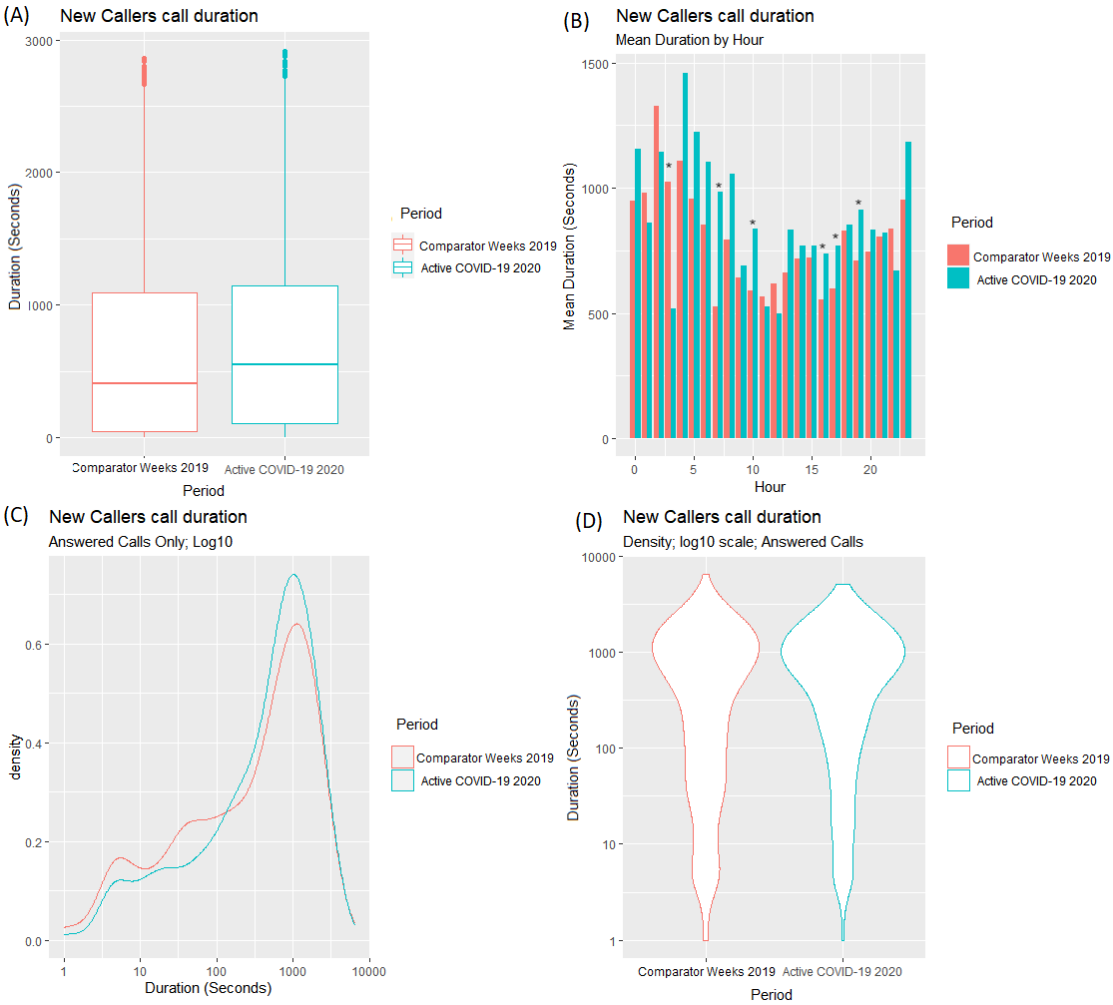
Differences in (A) call duration for answered calls by new callers in the Pre–COVID-19 and Active COVID-19 periods; (B) mean duration of calls by hour of day; and (C,D) density of call duration.

This section examines the behavior of callers who contacted the service for the first time in the Active COVID-19 period and do not appear in any other record within the data (as far back as January 1, 2019). For comparison, callers who contacted the service for the first time (again, since January 1, 2019) in weeks 14-17 in 2019 (the same time period in 2019 as the Active COVID-19 period in 2020) were also analyzed (see Figure 8). Both these cohorts are termed *new callers.*

Figure 8 displays the distributions of the call duration for answered calls in the comparative Weeks 14-17 in 2019 and the same weeks in 2020 (the Active COVID-19 period).

The mean and median call durations (Figure 8A) increased from the comparative 2019 period (mean=766 seconds/12.77 minutes; median= 450 seconds/7.5 minutes) to the Active COVID-19 period in 2020 (mean=831 seconds/13.85 minutes; median=582 seconds/9.7 minutes). A *t* test found a significant difference between periods with regards to call duration (t_3212_=–2.1943, *P*=.03).

## Discussion

### Principal Findings

The aims of this study were to examine which aspects of caller behavior to a national crisis helpline were impacted as a result of the COVID-19 pandemic and to determine which cohorts of callers were impacted the most. Call detail records spanning two periods of four weeks were compared; one period spanned the four weeks prior to the first confirmed case of COVID-19 in Ireland, and the other spanned the four weeks after the implementation of the restrictive lockdown by the Irish government. We also used k-means clustering to identify the types of callers who contacted the service based on their usage patterns. These data may help us understand which cohorts of callers required more support as a result of lockdown restrictions applied to manage the spread of the virus. Differences in call durations were analyzed for all callers, at a cluster level, and at a level that included “common callers” who had contacted the service in both time periods.

The findings suggest that caller behavior changed as a result of the COVID-19 pandemic and that due to the pandemic, callers made more calls of a longer duration and made fewer calls of a shorter duration. There were fewer calls around 5 minutes in duration and more calls lasting 30 minutes and longer. There were also differences in average duration at an hourly level between both periods, with a statistically significant difference in mean call duration in 22 of 24 hours. In the Active COVID-19 time period, average call durations peaked at around 3 AM and 4 AM (Figure 2). However, in the Active COVID-19 time period, while call durations across the day were greater for each hour, this increase was relatively small (approximately 3-5 minutes). Therefore, consideration must be given as to whether these differences at an hourly level are actually practically significant.

Differences in call duration were then analyzed on a week-by-week basis to determine if any progressive changes were evident. There was a variation in call duration density across the weeks in the Pre–COVID-19 period, meaning that the call duration varied from week to week. In contrast, within the Active COVID-19 weeks, very little variation in call duration density was observed, with no significant differences observed across these weeks. There was a trend toward more calls with longer durations and fewer calls with shorter durations across the Active COVID-19 weeks, with a sustained high density of longer duration calls across the four weeks at this time.

The analysis demonstrated significant differences in the behaviors of some of the caller types that were identified through clustering. There were changes within three particular cohorts of callers. High Frequency callers, Single Lengthy callers, and Typical callers, who previously all exhibited a mix of 5-minute and 30-minute calls, now tended to make longer calls. These changes appear to be more profound between the hours of 1 AM and 6 AM, where these callers spent much longer on the telephone on average.

Due to the lockdown restrictions, many of the existing mental health support groups have either had their services limited or have been removed altogether, particularly if these services relied on face-to-face appointments [6]. For instance, Samaritans Ireland halted all its face-to-face services from the commencement of the lockdown in Ireland but continued to offer telephone and email service [10], and the Health Service Executive of Ireland provided contact references for web-based and telephone support in the absence of face-to-face services [21]. These web-based and telephone services are solutions to the removal of face-to-face services and are considered to be effective in reducing levels of anxiety and depression [6,22]. These resources are vital at this time given that people are more likely to suffer from symptoms of mental illness, particularly anxiety and depression [2,3]. Callers who used the service because they already felt isolated may have an increased sense of isolation due to the restrictions imposed as a result of the pandemic. These findings attest to the impact of the pandemic on mental health and the need for additional support. They may well also reflect increased levels of distress amongst some high risk callers compared with the other caller types, such as the High Frequency callers, Single Lengthy callers, and Typical callers.

Suicide rates have been known to increase as a result of historical pandemics, such as the influenza epidemic in the United States between 1918 and 1919 [23] and the severe acute respiratory syndrome (SARS) epidemic [5,24]. Individuals with pre-existing mental illnesses will likely see their symptoms become exacerbated due to the pandemic. Furthermore, individuals with no pre-existing disorders may begin to develop a disorder, such as depression, anxiety, or posttraumatic stress disorder; these disorders may be more prevalent in essential and frontline health care workers, particularly medical personnel, due to the physical and emotional stress caused by extreme workloads and by experiencing traumatic events in the workplace [5].

There is agreement within the literature that many aspects of an individual’s daily routine may have changed as a result of the pandemic [5]. Individuals are staying at home and working from home or may have lost their jobs. People are experiencing reduced social interactions with others during this lockdown and are creating new routines to facilitate family needs. Home confinement has been stressful for many and has disturbed daily routines. Altena et al [25] summarized how confinement imposed by the COVID-19 pandemic can disrupt sleeping patterns by highlighting the factors that impact the stress-sleep link. Individuals who are more susceptible to stress-related sleep disruption are more likely to experience impacts to their circadian rhythms and develop insomnia as a result [25,26]. If the pandemic is causing people to feel more stressed and disrupts sleep and circadian rhythms as a result, this may explain why there has been a shift to a higher frequency of early morning calls, with the longer call durations reflecting daily routines that have been altered as a result of the pandemic.

### Future Research

One suggestion for future research is to analyze an additional period of data beyond the Active COVID-19 period to determine whether the aspects of caller behavior analyzed within this study returned to a Pre–COVID-19 period norm as a result of the easing of the lockdown restrictions. As new positive cases decline as a result of social distancing and lockdown measures, this may parallel a decrease in distress within the population, resulting in fewer emotional support calls being made to the service. If a decrease in the number of emotional support calls parallels the easing of lockdown restrictions, this may also represent habituation to a new norm caused by the pandemic or indicate that individuals have developed appropriate coping strategies to alleviate pandemic-related distress [27].

### Implications for Policy and Practice

These findings provide an indication of the impact of the pandemic on the behavior of people who use crisis line services. They may also point to the need for high risk individuals to have increased support to mitigate the impact of the virus and measures taken to minimize spread on their well-being and mental health. The changes in the times that people used the service can inform service planning and volunteer scheduling to ensure that more calls could be answered at the new peak times. Although this was not directly assessed within the current study, the increase in the trend toward longer calls being made to the service may have taken up more service capacity. If this is the case, it may be necessary to alter volunteer scheduling to meet this change in demand. Volunteers may have to undergo retraining to prepare for new presenting reasons associated with the pandemic, such as bereavement due to COVID-19, becoming unemployed or furloughed, and increased isolation. Lastly, as call durations have increased over the Active COVID-19 period, callers may require further training in preparation for longer conversations.

This work highlights the need for mental health and well-being and suicide prevention support services, such as crisis helplines, to be provided with the appropriate support and funding to mitigate the impact of the pandemic on the mental well-being of the population. If services such as crisis helplines are funded appropriately, this may also help prevent further worsening of mental health within the population and thus relieve strain on national health services [28,29]. It is important to recognize that while the reproductive rate of COVID-19 is gradually decreasing over time, there is a possibility that the virus can return as a second wave or local outbreak. The findings in this study may be used to inform similar services of how the behavior of their clients may change as a result of the pandemic, which affords services the opportunity to change aspects of their service to mitigate the impact of the pandemic.

### Limitations

There are some inherent limitations to consider when interpreting this type of call data. Each caller who contacts the service is represented in the call data by an anonymized identifier based on the telephone number used. It is not possible to know whether the same telephone has been used by multiple individuals to contact the service. This may be the case in a small minority of cases and also in residential settings, and it would lead to misclassification of that caller. In addition, callers who had insufficient contact with the service to accurately classify them as high-frequency users may have been misclassified by the clustering algorithm [17].

One limitation of the current study is that no demographic information about the callers was available for analysis. The Samaritans Ireland service is entirely confidential. Volunteers may ask for the name of the caller, as this is a natural element of conversation; however, callers can remain anonymous if they wish. Moreover, caller demographic information was not available for analysis. However, if caller demographic information were available for analysis, it may be of interest to learn which cohorts of the population were most likely to contact the service during both periods within the study and determine which demographic was most impacted.

### Conclusions

This study investigated the impact of the COVID-19 pandemic on aspects of the behavior of callers to Samaritans Ireland, a national crisis helpline. Aspects of behavior from callers who contacted the service within two comparable time periods, a Pre–COVID-19 period and an Active COVID-19 period, were analyzed.

Visible differences were observed in caller behavior from the Pre–COVID-19 to Active COVID-19 periods. Callers made fewer calls of a short duration and trended toward making more calls of a longer duration. Callers also appeared to make longer calls across all but one hour of the day. At a weekly level, the density of call durations was highly variable across the four individual weeks within the Pre–COVID-19 period. In contrast, the density of call durations was highly stable with very few differences between each individual week within the Active COVID-19 period. Moreover, callers trended toward making more and longer calls to the service across the four individual weeks in the Active COVID-19 period. At a cluster level, there were statistical differences between three of five caller types in relation to call duration density; these callers trended toward fewer shorter calls and more longer calls. Changes in the mean duration were observed at hourly intervals, with the most pronounced changes between the hours of 1 AM and 6 AM.

This work provides evidence of the impact of the COVID-19 pandemic on mental well-being within the population and its impact on support-seeking and help-seeking behavior. The patterns identified in this research suggest that callers have additional mental health and suicide prevention support needs as a result of the effects of the pandemic and that helplines can play a vital role in helping to meet these needs.

## Abbreviations

API: application programming interface
PHEIC: Public Health Emergency of International Concern
SARS: severe acute respiratory syndrome
WHO: World Health Organization
